# Prior Exercise Attenuates LPS-Induced Neuroinflammation, Dopaminergic Dysfunction, and Fatigue-like Behavior in Mice

**DOI:** 10.1021/acsomega.5c05266

**Published:** 2025-11-18

**Authors:** Ana Cristina de Bem Alves, Naiara de Souza Santos, Ananda Christina Staats Pires, Ana Elisa Speck, Tatyana Nery, Amanda Leite Bastos-Pereira, Débora da Luz Scheffer, Rui Daniel Prediger, Alexandra Latini, Aderbal Silva Aguiar

**Affiliations:** † Biology of Exercise Lab (Labioex), Department of Physical Therapy, UFSC-Federal University of Santa Catarina, 88905-120 Araranguá, SC, Brazil; ‡ Laboratory of Bioenergetics and Oxidative Stress (Labox), Department of Biochemistry, UFSC-Federal University of Santa Catarina, 88040-900 Florianópolis, SC, Brazil; § Experimental Laboratory of Neurodegenerative Diseases (LEXDON), Department of Pharmacology, Federal University of Santa Catarina, 88040-900 Florianópolis, SC, Brazil

## Abstract

*Purpose*: Neuroinflammation contributes to central fatigue by disrupting dopaminergic signaling and motivational circuits. This study investigated whether prior voluntary exercise attenuates behavioral, physiological, and neurochemical alterations induced by systemic inflammation. *Methods*: Adult male Swiss mice (8–10 weeks old, 35–40 g) were assigned to sedentary or runner groups. with 6 weeks of free wheel running. Systemic inflammation was induced via intraperitoneal injections of lipopolysaccharide (LPS, 0.33 mg/kg, *Escherichia coli*), while controls received saline (0.9% NaCl). Behavioral assessments included locomotion, grooming, and social interaction. Fatigue-related outcomes were evaluated via maximal oxygen consumption (V̇O_2_max) and grip strength. Prefrontal cortex IL-1β and IL-6 levels were quantified by ELISA, and dopaminergic metabolism was assessed by HPLC. *Results*: LPS exposure elicited marked neuroinflammatory responses—elevated IL-1β and IL-6—and disrupted dopamine metabolism in the prefrontal cortex. These changes correlated with impaired behavior, reduced V̇O_2_max, and diminished grip strength. Prior exercise attenuated IL-6 elevation, preserved dopaminergic markers, and mitigated some behavioral and functional deficits. However, IL-1β levels remained elevated and protection was incomplete. *Conclusion*: Systemic inflammation is associated with impaired CNS function, reflected by neuroinflammatory changes and alterations in dopaminergic metabolism. Prior physical activity confers partial protection, reducing IL-6 and preserving behavioral and metabolic performance. These findings highlight the potential of exercise as a preventive strategy against inflammation-induced central fatigue.

## Graphical abstract

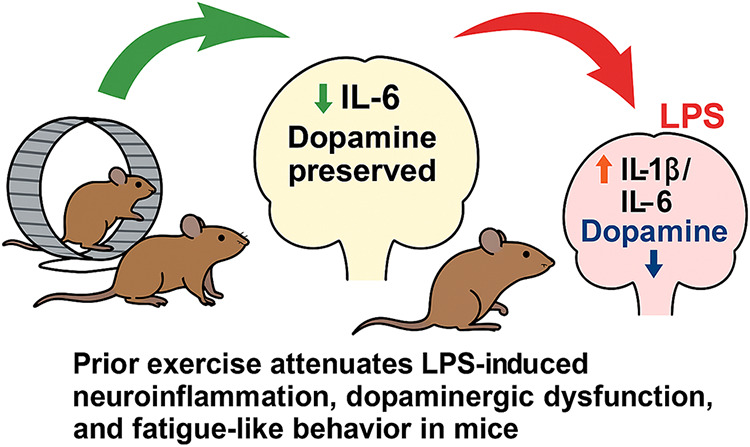

## 1 Introduction

Chronic inflammation is a pathological condition characterized by persistent immune activation and systemic physiological disruptions.^1–3^ This state is commonly associated with symptoms such as fatigue and malaise, which significantly impact quality of life, particularly in individuals with autoimmune^4–6^ and neurodegenerative diseases.^7–10^ Neuroinflammation, characterized by increased levels of markers such as Interleukin-1 β (IL-1β) and Interleukin-6 (IL-6), can disrupt key central nervous system (CNS) processes, including neurotransmission,^11–15^ neuromodulation,^11,16–19^ neuroinflammation,^20–23^ dopaminergic signaling, and cerebralmetabolism,^19,24^ particularly in regions such as the prefrontal cortex.

Fatigue is a prominent symptom in conditions associated with chronic inflammation, yet its underlying mechanisms remain incompletely understood. While peripheral fatigue, resulting from muscle fatigability and neuromuscular dysfunction, has been extensively studied using objective measures such as the decline in force during voluntary contractions,^2,3,9^ central fatigue—which encompasses cognitive, motivational, and behavioral components—has been linked to neuroinflammation-driven disruptions in brain function. The distinction is crucial, as central fatigue is often associated with alterations in motivational circuits, dopaminergic tone, and inflammatory cytokine signaling, while peripheral fatigue reflects local muscle physiology. Inflammatory cytokines such as IL-1β and IL-6 play a role in neuroimmune communication and have been implicated in sickness behavior, motivational deficits, and cognitive impairments observed in inflammatory conditions.^20–23^ However, the extent to which neuroinflammatory alterations drive these behavioral changes, and whether interventions such as physical exercise can mitigate these effects, remains an open question.

Accumulating evidence suggests that physical exercise exerts anti-inflammatory and neuroprotective effects, which could counteract inflammation-driven fatigue. Exercise performed prior to an inflammatory challenge has been shown to reduce neuroinflammatory responses by modulating glial activation, suppressing excessive cytokine release, and enhancing neurotrophic factor expression, including FNDC5 and brain-derived neurotrophic factor (BDNF).^25–27^ Experimental models demonstrate that preconditioning with aerobic training before inflammation is induced enhances neurogenesis, preserves mitochondrial function, and protects dopaminergic circuits from inflammation-induced damage.^28–33^ Nevertheless, most studies have not evaluated whether these adaptations translate into protection against functional outcomes such as aerobic capacity, neuromuscular performance, and motivation-related behaviors. Furthermore, the relationship between V̇O_2_max—a widely accepted marker of aerobic performance and central fatigue—and neuroinflammation remains poorly explored.

In light of these gaps, we designed the present study to test whether prior voluntary aerobic exercise confers resilience against behavioral, physiological, and neurochemical impairments triggered by systemic inflammation. We employed a six-week protocol of free wheel running in male Swiss mice, followed by an acute LPS challenge, to model systemic inflammation and investigate central fatigue. Specifically, we assessed sickness behavior, VO_2_max, grip strength, and neuroinflammatory and dopaminergic markers in the prefrontal cortex. By integrating behavioral and biochemical analyses, we aimed to determine whether prior exercise mitigates the impact of systemic inflammation on fatigue-related outcomes.

It is important to note that this study is descriptive and does not establish causal relationships between neuroinflammatory markers, dopamine metabolism, and behavioral outcomes. While our use of an acute LPS model in healthy young mice allows for a controlled investigation of central fatigue mechanisms, it does not fully replicate the chronic and multifaceted nature of fatigue observed in conditions such as major depressive disorder or autoimmune diseases. However, by linking prior physical activity to specific neurochemical and behavioral adaptations under inflammatory stress, this study offers novel evidence for the role of exercise as a preemptive, nonpharmacological intervention to mitigate central fatigue.

## 2 Methods

### 2.1 Animals–Sourcing, Housing, and Exercise Group Allocation

Ninety-one adult male Swiss mice (8–10 weeks old, 35–40 g) were obtained from the Central Animal Facility of the Federal University of Santa Catarina (UFSC). All procedures were approved by the Institutional Animal Care and Use Committee (IACUC PP10616; CEUA3552210222). Animals were group-housed (3–5 per cage) in polypropylene cages (38 cm × 32 cm × 17 cm) with wood shavings, maintained in ventilated cabinets under controlled conditions (12 h light/dark cycle, lights on at 07:00, temperature 21 ± 1 °C), with ad libitum access to food (Nuvilab CR1, 3.9 kcal/g) and filtered water.

The experimental design is illustrated in Figure 1. Behavioral and fatigue experiments were performed in distinct cohorts to avoid confounding effects. Mice were randomly assigned to sedentary (SED) or exercise (RW) groups. RW mice were housed individually (27 cm × 18 cm × 13 cm) with operational 4.5-in. running wheels (Super Pet, USA) equipped with magnetic sensors and digital counters to monitor wheel revolutions,^34–36^ while SED mice were housed in identical cages with locked (nonrotating) wheels to control for environmental enrichment. Running distances were recorded daily at 19:00. Randomization was conducted using a computer-generated sequence, and each mouse was treated as an independent experimental unit.

**Figure 1:**
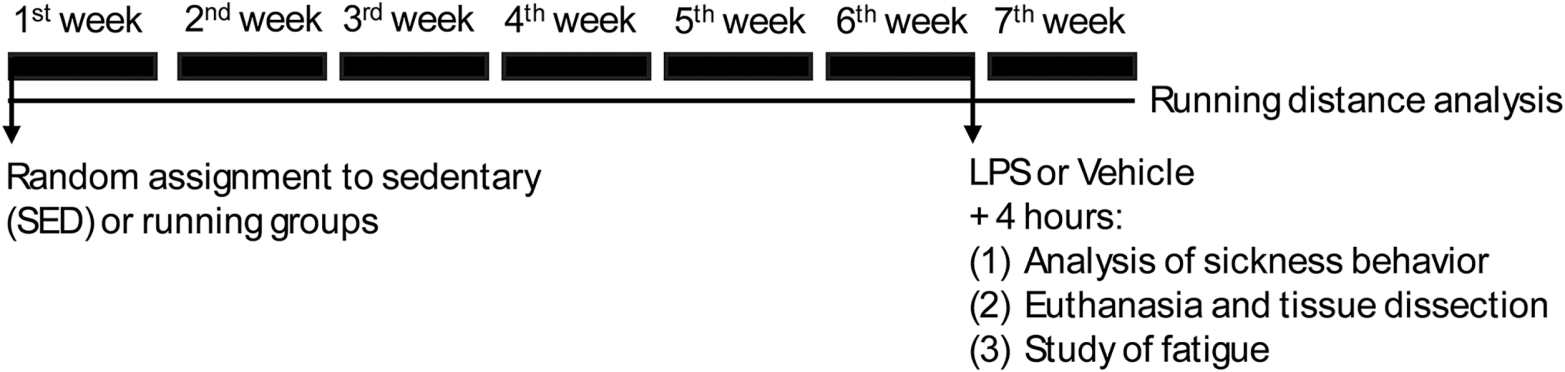
Experimental design: Researchers randomly assigned mice to either sedentary or running wheel (RW) groups. After 6 weeks, the mice were randomly allocated to receive either LPS (at a specified dose) or vehicle treatments. Four hours post-treatment, researchers assessed fatigue and sickness behaviors before euthanizing the mice for muscle and brain dissection for biochemical analysis. In a separate experiment, another group of mice continued running for an additional week to evaluate the impact of LPS on wheel performance. All experiments utilized independent groups of animals.

### 2.2 Postexercise LPS Treatment: Analysis of Fatigue and Sickness Behavior

After 6 weeks of voluntary exercise, both RW and SED mice were randomly assigned to receive an intraperitoneal (i.p.) injection of lipopolysaccharide (LPS; 0.33 mg/kg, *E. coli* 0127:B8, Sigma) or saline (0.9% NaCl) between 09:00 and 11:00. This resulted in four experimental groups: SED-SAL, SED-LPS, RW-SAL, and RW-LPS. All injections were administered under similar ambient conditions to minimize circadian or environmental variability.

All subsequent behavioral and fatigue assessments were conducted between 13:00 and 15:00, 4 h postinjection, a time point known to elicit a transient but robust neuroimmune response in mice.^37,38^ Importantly, independent cohorts were used for behavioral, physiological, and biochemical analyses to avoid confounding due to prior test exposure. A subset of mice had wheel activity continuously monitored for one additional week post-LPS to assess ongoing performance.

#### 2.2.1 Metabolic Exercise Test

To assess aerobic fatigue, mice were acclimated over 3 days to a treadmill (Bonther, Ribeirão Preto, SP, Brazil). The acclimation protocol included: 10 min at 5 m/min (day 1), 5 min at 5 m/min and 5 min at 10 m/min (day 2), and 10 min at 10 m/min (day 3). Following a 48 h rest, mice underwent incremental ergospirometry, starting at 5 m/min and increasing by 5 m/min every 3 min, on a 1.7° incline with 0.2 mA stimulus. Oxygen consumption (V̇O_2_) and carbon dioxide production (V̇CO_2_) were recorded in a metabolic chamber attached to the treadmill. Respiratory Exchange Ratio (RER = V̇CO_2_/V̇O_2_) and vertical running power were also calculated.^17,18,39^ The primary criterion for maximal exertion was RER ≥ 1.0.^17,18,40^ As secondary indicators, experimenters monitored for a plateau tendency in V̇O_2_ curves and for exhaustion-related behaviors (e.g., consistent refusal to continue running despite mild stimulus). These complementary signs were not used as formal end points but provided supportive information. Blood lactate was not assessed because construction of lactate curves would require serial sampling and a larger number of animals, which was not compatible with IACUC guidelines focused on refinement and reduction. Animals used for this test were not subjected to biochemical procedures.

#### 2.2.2 Grip Force Meter

To assess anaerobic fatigue, we used the Bonther 5 kgf grip force meter (Bonther, Ribeirão Preto, SP, Brazil) for forelimb grip strength measurement. Each mouse was positioned on the apparatus bar, and its tail was gently pulled to counteract its front paws’ grip for four 10 s trials. In our study, the vertical version^41^ of the test was employed, where the gauge was rotated vertically and fixed to a metal stand to immobilize the system. This setup allowed for consistent measurements while ensuring that the animal’s movements did not interfere with the results. The peak pull force (in gram-force, gf) and grip duration were calculated automatically using the software accompanying the equipment.^11,19,42^ To ensure accuracy, trials in which only one forepaw was used, where the mouse turned during the pull, or when it left the bar without resistance, were excluded from analysis. The pulling speed was standardized across all trials, performed at a sufficiently slow and consistent rate to allow mice to build resistance before releasing the bar. To mitigate fatigue effects within sessions, the order of mice tested each day was randomized, and all test sessions were conducted during the afternoon hours of the light cycle (11 AM to 5 PM). The average of the top three trial strengths was calculated as the grip strength score.^41,43^ Impulse was determined as the cumulative force exerted over time during the grip test, with the area under the curve (AUC) computed from force-time curves, providing an integrated measure of muscle performance.^11,42^ These metrics, calculated using the equipment’s software, offer a comprehensive evaluation of muscular effort beyond peak force. Mice subjected to grip strength testing were not included in subsequent biochemical analyses to prevent potential confounding factors related to physical exertion.

#### 2.2.3 Open Field Test

Mice were individually placed at the center of a 60 cm diameter circular arena under dim light (≈10 lx) and allowed to move freely for 5 min. The arena was cleaned with 20% alcohol between each session. We assessed motor function by recording distance traveled and rearing events using Stoelting Co.’s ANY-maze software.

#### 2.2.4 Social Interaction Test

Following habituation to the open field context, a novel female mouse in natural estrous cycle was introduced into the same arena for a 5 min interaction period. Each male was tested with a different female to ensure unbiased results. Social behaviors including approaching, following, and sniffing^44,45^ were manually timed using Stoelting Co.’s ANY-maze software. Specific keys were configured within the software to record the duration of each social behavior in real time, with the experimenter pressing the corresponding key during each event. All analyses were performed by an experimenter blind to the experimental conditions to minimize bias.

The estrous cycle of the conspecific female was not synchronized between groups, which we acknowledge as a potential source of variability. However, the short test duration and the nature of the scored parameters (approach, following, sniffing) suggest that the task predominantly reflects social motivation rather than copulatory behavior, thereby reducing the influence of estrous variability. This limitation is further addressed in the Section 4.4.

#### 2.2.5 Splash Test

The Splash test, conducted after the open field test, evaluates self-care behavior by monitoring grooming activities following a 10% sucrose solution application to the animal’s back.^46^ Grooming behaviors—including nose/face grooming, head washing, and body grooming^47^—were video-recorded for 5 min in the open field arena under dim light (≈10 lx) and quantified using Stoelting Co.’s ANY-maze software. Both grooming frequency and cumulative grooming duration were analyzed, as these parameters are widely interpreted as indicators of self-care and motivational drive. In our study, reduced grooming was considered a motivational deficit, but always interpreted together with locomotor data to avoid misattribution to motor impairment. Each animal was tested individually, and the arena was cleaned with 20% alcohol between sessions.

##### 2.2.5.1 Terminology and Construct Definitions

In this study, sickness behavior refers to the acute constellation of LPS-induced responses, including reduced locomotion and grooming. Fatigue-like behavior is defined by impaired physiological performance (reduced V̇O_2_max and grip strength). Motivational deficits are inferred from reductions in grooming and social interaction, but always interpreted in the context of locomotor activity. These operational definitions are applied consistently across behavioral tests and figures.

Immediately after each behavioral test, mice were euthanized for blood and whole prefrontal cortex collection. Blood was centrifuged at 5000 rpm for 5 min at 4 °C to isolate serum, which was stored at −80 °C for cytokine quantification.

### 2.3 Biomarker Quantification–ELISA and HPLC Analyses

Enzyme-Linked Immunosorbent Assay (ELISA). The prefrontal cortex was homogenized in ice-cold buffer (pH 7.4) containing 10 mM Tris, 1 mM EDTA, 1% Triton, and protease inhibitors (aprotinin, chemostatin, leupeptin, and phenylmethylsulfonyl fluoride, each at 1 μg/mL). The homogenate was centrifuged at 14,000 rpm for 10 min at 4 °C, and the resulting supernatant was collected for cytokine quantification.

IL-1β and IL-6 levels were measured using ELISA kits according to the manufacturer’s instructions. For IL-1β, the Quantikine Mouse IL-1β Immunoassay Kit (R&D Systems, MLB00C) was used (sample dilution 1:5); for IL-6, the DuoSet ELISA Development Kit (R&D Systems, DY406) was used (sample dilution 1:10). Cytokine concentrations were interpolated from standard curves measured colorimetrically at 450 nm, with a correction at 540 nm to adjust for optical imperfections, using an Infinite 200 PRO ELISA reader (TECAN, Switzerland). The assay sensitivities were 2.31 pg/mL (IL-1β) and 15.6 pg/mL (IL-6), with intra- and interassay coefficients of variation below 10%. Results are expressed as picograms per milliliter (pg/mL).

High-Performance Liquid Chromatography (HPLC). The prelimbic, infralimbic, anterior cingulate, and orbitofrontal regions of the prefrontal cortex were dissected bilaterally (see Supporting Figure 1), weighed individually (10–15 mg), and immediately frozen at −80 °C. Tissues were homogenized in 0.1 N perchloric acid containing 0.02% sodium metabisulfite (1:10, w/v), sonicated, and centrifuged at 16,000*g* for 10 min at 4 °C. The supernatant (20 μL) was injected into an HPLC system (Alliance e2695, Waters, MA, USA) coupled to an electrochemical detector (Waters 2465, +400 mV). Separation was performed using a 150 mm × 2.0 mm, 4 μm C18 reverse-phase column (Synergi Hydro, CA, USA) maintained at 35 °C.

The mobile phase consisted of 90 mM sodium phosphate, 50 mM citric acid, 1.7 mM sodium heptanesulfonate, 50 μM EDTA, and 10% acetonitrile, adjusted to pH 3.0, and was delivered at 0.25 mL/min. Dopamine (DA) and its primary metabolite 3,4-dihydroxyphenylacetic acid (DOPAC) were quantified using external standard calibration and normalized to protein content (ng/mg protein).

### 2.4 Statistics

All data are expressed as mean ± standard error of the mean (SEM) and were analyzed using GraphPad Prism 10 (GraphPad Software, USA) and STATISTICA 13.5.0.17 (StatSoft, Inc., USA). Behavioral and fatigue-related data were analyzed by two-way ANOVA followed by Tukey’s *post hoc* test, with factors being physical activity (SED vs RW) and treatment (SAL vs LPS). Repeated-measures ANOVA with Bonferroni’s *post hoc* test was applied to assess running distance across days and V̇O_2_ as a function of running power. RER values were compared to the fatigue threshold (RER = 1.0) using a one-sample *t* test. The significance level was set at *p* < 0.05 for all comparisons. Descriptive statistics and individual *F* values are reported in the Section 3.

### 2.5 Data Availability Statement

This work is available as open data under the Creative Commons Attribution (CC BY) license.^48^

## 3 Results

Mice subjected to the six-week running regimen exhibited a progressive increase in daily distance, as shown by a significant main effect of time (*F*_41,8_ = 3.8, *p* < 0.05, Figure 2A). Bonferroni post hoc comparisons confirmed a significant increase in distance between the first and last weeks of training. One week after LPS administration, running performance declined significantly (*F*_5,1_ = 4.4, *p* < 0.05), with Bonferroni *post hoc* tests revealing a reduction compared to pre-LPS levels.

**Figure 2:**
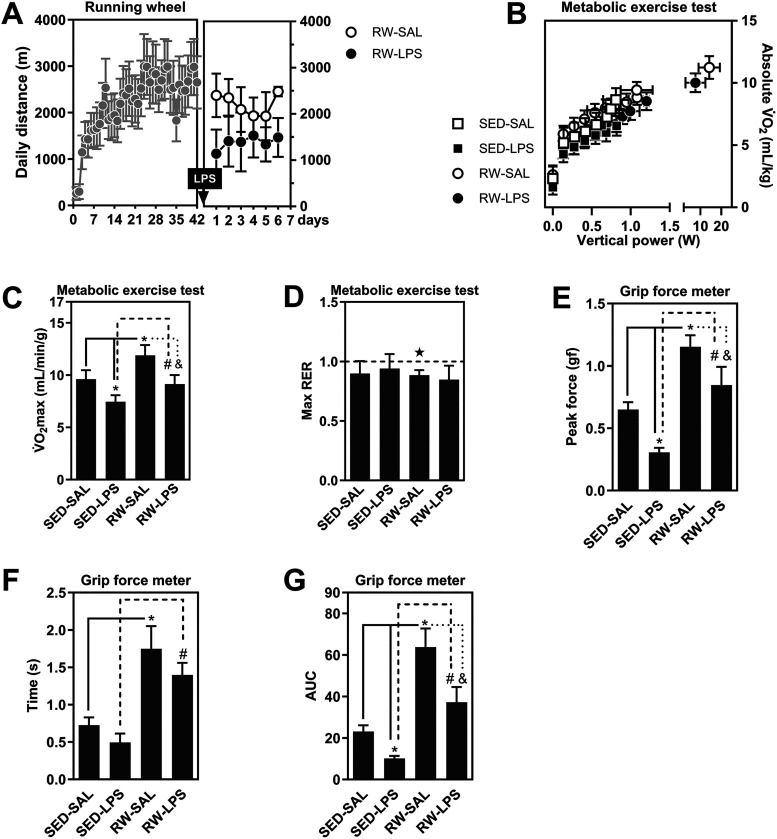
Impact of exercise and LPS on physiological performance (fatigue-like behavior). This figure shows the effects of a six-week running regimen and LPS administration on aerobic and anaerobic performance. Running increased daily distance, whereas LPS reduced it (A). Exercise improved oxygen consumption, but this enhancement was impaired by LPS (B–C). All animals, except saline-treated runners, reached an RER of 1.0, indicating maximal exertion (D). Exercise boosted grip strength and impulse, while LPS counteracted these gains (E–G). Data are mean ± standard error of the mean. *N* = 8–10 animals/group for 9–10 independent experiments. Group comparisons (SED-SAL, SED-LPS, RW-SAL, RW-LPS) were analyzed by two-way ANOVA followed by *Tukey’s post hoc* test. Significance is indicated as *p* < 0.05 versus SED-SAL (*), SED-LPS (#), or RW-SAL (&). For RER data, significance versus 1.0 was determined by a one-sample *t*-test (★).

During the metabolic exercise test, LPS administration impaired both submaximal oxygen consumption (*F*_6,150_ = 81.4, *p* < 0.05, Figure 2B) and maximal oxygen consumption (V̇O_2_max; *F*_3,27_ = 9.4, *p* < 0.05, Figure 2C). Tukey’s *post hoc* analysis showed that LPS significantly reduced V̇O_2_max in sedentary mice, whereas this effect was attenuated in the exercised group, indicating partial preservation of aerobic capacity. Due to overlapping curves in Figure 2B, individual group trajectories are detailed in Supporting Figure 2.

As shown in Figure 2D, RER values at exhaustion were statistically equal to 1.0 across most groups, except for exercised mice treated with saline (*t*_7_ = 2.7, *p* < 0.05), as determined by one-sample *t* test, suggesting greater metabolic reserve and the potential to continue running beyond the fatigue threshold.

LPS significantly reduced grip strength (*F*_3,18_ = 10.5 *p* < 0.05, Figure 2E) and impulse (*F*_3,18_ = 8.9, *p* < 0.05, Figure 2G). Tukey’s *post hoc* tests revealed that these deficits occurred in sedentary LPS-treated mice but were mitigated in the exercised group, supporting a protective effect of prior physical activity. Moreover, exercise significantly increased grip duration (*F*_3,18_ = 22.4, *p* < 0.05, Figure 2F), as confirmed by Tukey’s *post hoc*, and this enhancement was not affected by LPS treatment, indicating improved neuromuscular endurance in physically active animals.

The animal model used induces sickness behavior,^36,37^ a phenotype observed in this study. LPS significantly reduced locomotion (*F*_3,28_ = 11.08, *p* < 0.05, Figure 3A) and rearing (*F*_3,28_ = 26.4, *p* < 0.05, Figure 3B) in both sedentary (SED) and exercised (RW) animals in the open field test. Tukey’s *post hoc* analysis revealed that rearing behavior was significantly higher in RW-LPS mice compared to SED-LPS, indicating a partial preservation of exploratory activity by prior exercise.

**Figure 3:**
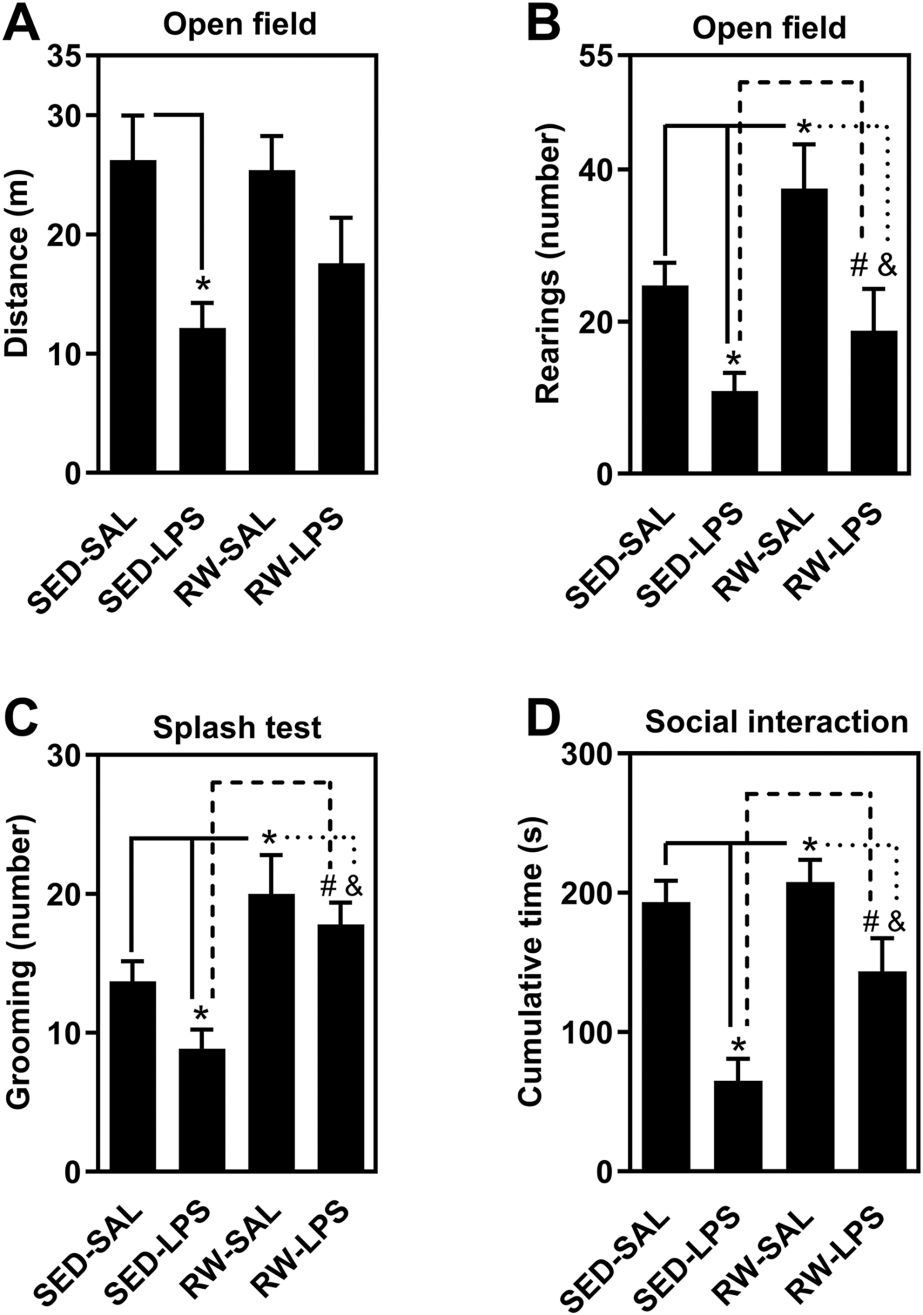
Impact of LPS and exercise on behavioral outcomes (sickness behavior and motivational deficits). This figure illustrates the effects of LPS administration on behavior, showing reduced locomotion and rearing in the open field test (A–B). Exercise mitigated these effects, particularly enhancing rearing behavior. LPS also induced sickness behavior (reduced grooming) and motivational deficits (decreased social engagement), both of which were attenuated by prior exercise (C–D). Data are presented as mean ± SEM *N* = 8–10 animals/group for 9–10 independent experiments. Group comparisons (SED-SAL, SED-LPS, RW-SAL, RW-LPS) were analyzed by two-way ANOVA followed by *Tukey’s post hoc* test. Significance is indicated as *p* < 0.05 versus SED-SAL (*), SED-LPS (#), or RW-SAL (&).

Additionally, LPS significantly reduced grooming behavior (*F*_3,28_ = 15.1, *p* < 0.05, Figure 3C) and social interaction time (*F*_3,28_ = 27.2, *p* < 0.05, Figure 3D). Tukey’s *post hoc* tests confirmed that physical activity significantly counteracted these deficits, with RW-LPS mice showing increased grooming and social engagement compared to SED-LPS mice. These findings support a protective behavioral effect of prior voluntary exercise.

LPS also induced marked neuroinflammatory changes and altered dopaminergic neurotransmission in the prefrontal cortex. Serum IL-1β levels were elevated (*F*_3,19_ = 8.8, *p* < 0.05, Figure 4A), and prefrontal cortex IL-1β was significantly increased by LPS (*F*_3,19_ = 8.9, *p* < 0.05, Figure 4B). Tukey’s post hoc analysis showed that IL-1β levels in SED-LPS mice were significantly higher than in SED-SAL mice, while exercise failed to significantly reduce IL-1β levels in LPS-treated animals, indicating only marginal anti-inflammatory effects on this cytokine.

**Figure 4:**
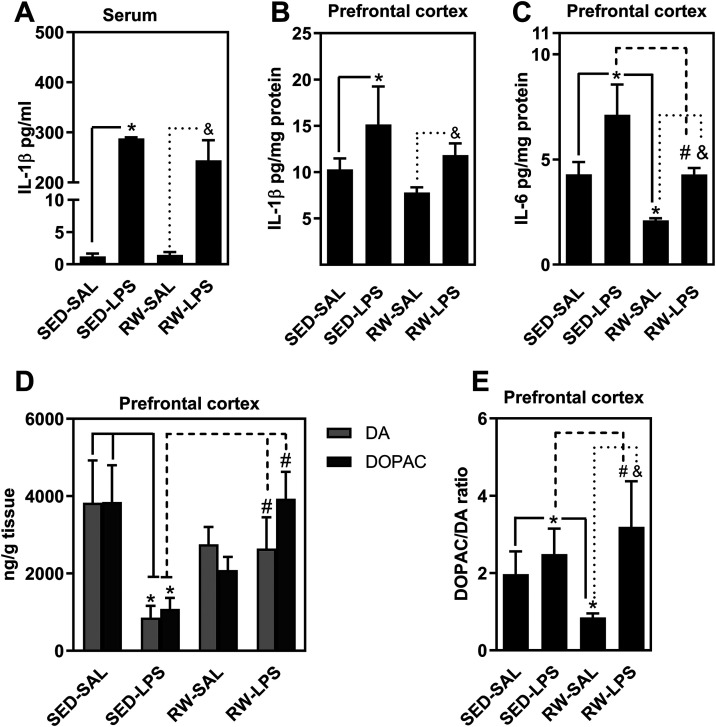
Impact of LPS and exercise on cytokine and dopaminergic alterations. The figure shows that LPS administration elevated IL-1β serum levels compared to saline-treated controls (A). In the prefrontal cortex, LPS increased IL-1β and IL-6 levels (B–C), while exercise partially reduced the IL-6 increase. LPS also reduced dopamine and DOPAC levels in the prefrontal cortex, effects that exercise fully prevented (D). Finally, LPS enhanced dopaminergic turnover (DOPAC/DA ratio), with exercise providing partial protection (E). Data are presented as mean ± SEM *N* = 8–10 animals/group for 9–10 independent experiments. Group comparisons (SED-SAL, SED-LPS, RW-SAL, RW-LPS) were analyzed by two-way ANOVA followed by *Tukey’s post hoc* test. Significance is indicated as *p* < 0.05 versus SED-SAL (*), SED-LPS (#), or RW-SAL (&).

Similarly, LPS significantly increased IL-6 levels in the prefrontal cortex (*F*_3,19_ = 8.8, *p* < 0.05, Figure 4C). Tukey’s *post hoc* tests indicated higher IL-6 levels in SED-LPS mice compared to SED-SAL, with a significant reduction in IL-6 levels in RW-LPS mice compared to SED-LPS, demonstrating a partial anti-inflammatory effect of exercise on this marker.

Moreover, LPS significantly decreased dopamine (DA) levels (*F*_3,24_ = 9.4, *p* < 0.05) and DOPAC (*F*_3,27_ = 11.2, *p* < 0.05) in the prefrontal cortex (Figure 4D). Tukey’s *post hoc* analysis confirmed reductions in DA and DOPAC in SED-LPS compared to SED-SAL mice, while RW-LPS animals had DA and DOPAC levels restored to those of saline-treated groups, indicating that exercise fully preserved dopaminergic tone.

Finally, LPS increased the DOPAC/DA ratio, reflecting enhanced dopaminergic turnover (*F*_3,25_ = 9.9 *p* < 0.05, Figure 4E). Tukey’s *post hoc* showed significant increases in SED-LPS compared to SED-SAL, and exercise partially prevented this elevation in RW-LPS mice.

## 4 Discussion

### 4.1 Neuroinflammation, Dopaminergic Alterations, and Central Fatigue

Central fatigue is a multifactorial condition distinct from peripheral fatigue, involving neuroimmune and neurotransmitter mechanisms rather than neuromuscular dysfunction.^12,49–51^ Cytokines such as IL-1β and IL-6 play key roles in sickness behavior and motivational deficits,^21,23^ and dopaminergic dysfunction has been implicated in effort-related processes.^8,52^ In our study, LPS administration produced robust behavioral and physiological alterations, including reduced locomotion, decreased V̇O_2_max and grip strength, and altered dopamine metabolism in the prefrontal cortex.^53–61^ These findings support an association between neuroinflammatory signaling and dopaminergic disruption underlying fatigue-like outcomes. Nevertheless, causality was not established, and future studies should employ pharmacological or genetic manipulations to clarify these pathways.

Prior exercise partially attenuated these alterations, reducing IL-6 elevation, preserving dopaminergic tone, and mitigating behavioral impairments. IL-1β remained elevated despite training, indicating that exercise does not uniformly suppress neuroinflammation. This selective modulation suggests distinct cytokine regulation and highlights IL-1β as a more stable marker of inflammation-induced fatigue.

### 4.2 Exercise, Cytokines, and Behavioral Outcomes

Exercise preserved aerobic and neuromuscular performance in the face of systemic inflammation, yet methodological limitations must be acknowledged. V̇O_2_max was estimated using only RER ≥ 1.0, which is insufficient without VO_2_ plateau or lactate end points; thus, these results should be interpreted cautiously.^40^ Moreover, all assessments occurred 4 h post-LPS, a time point dominated by acute sickness. Consequently, behavioral deficits may reflect malaise rather than chronic fatigue, as also noted by Reviewer 2. Grooming and social interaction are secondarily dependent on locomotion,^47^ and we now emphasize this confounding factor.

Our cytokine analysis was limited to IL-1β and IL-6, which restricts conclusions about global “attenuation of neuroinflammation.” Exercise lowered IL-6 but not IL-1β, suggesting a selective adaptation. Future studies should expand cytokine profiling to include TNF-α, IL-10, and others. The protective effects of training may also involve neurotrophic factors such as BDNF and systemic immune modulation,^25,36,62,63^ although these were not directly assessed here.

### 4.3 Dopamine and Adenosine Modulation in Central Fatigue

Dopamine is central to motivation, motor control, and reward.^11,13,19,42,52,64,65^ LPS-induced reductions in dopamine and DOPAC in the prefrontal cortex, prevented by exercise, suggest dopaminergic vulnerability to inflammatory insults. The DOPAC/DA ratio should be interpreted as an indirect marker of altered dopamine turnover^66,67^ , rather than as a definitive measure of metabolism, since direct markers of synthesis and reuptake (TH, DAT, COMT) were not assessed.

In addition, adenosine A_2_A receptors may represent an important mechanistic link between inflammation, dopamine, and motivational behavior. A_2_A receptors modulate dopaminergic function in the prefrontal cortex^69,70^ and influence cytokine signaling.^71–73^ Long-term exercise also modulates striatal A_2_A receptors and has been associated with protective adaptations against central fatigue.^68^ Although not assessed here, this pathway provides a promising direction for future studies aiming to integrate neuroinflammatory and neuromodulatory mechanisms of central fatigue.

### 4.4 Limitations and Perspectives

his study has several limitations: (i) only male mice were used, limiting generalizability across sexes;^39^ (ii) behavioral and biochemical measures were obtained in separate cohorts, precluding individual-level correlations; (iii) cytokine analysis was restricted to IL-1β and IL-6; (iv) V̇O_2_max was estimated primarily by RER ≥ 1.0, complemented by visual inspection of V̇O_2_ curves and exhaustion-related behaviors, but without lactate measurements;^40^ (v) the acute LPS model may not fully capture chronic fatigue syndromes; (vi) prefrontal cortex dissections pooled prelimbic, infralimbic, anterior cingulate, and orbitofrontal regions bilaterally, which precludes region-specific or lateralized analyses; and (vii) the social interaction test employed females in natural estrous cycle without synchronization, which may introduce variability. However, the short test duration and the nature of the recorded behaviors (approach, following, sniffing) suggest that social motivation, rather than copulatory behavior, was the primary factor influencing responses. Future studies could standardize estrous stages or adopt same-sex interaction protocols to minimize this potential confound. Despite these constraints, our findings demonstrate that prior exercise attenuates specific neuroinflammatory and dopaminergic alterations associated with systemic inflammation, supporting physical activity as a preventive strategy against central fatigue.

## 5 Conclusion

This study demonstrates that LPS-induced neuroinflammation contributes to central fatigue, characterized by impaired performance and altered dopaminergic metabolism in the prefrontal cortex. Prior exercise partially prevented these effects, modulating cytokine levels and preserving function. These findings highlight exercise as a potential preventive strategy against inflammation-induced central fatigue.

## Supplementary Material



## Notes

Prefrontal cortex dissection areas (Figure S1) and VO_2_ group curves from the incremental treadmill test (Figure S2) (PDF)
