# Supplementary material for: Prior Exercise Attenuates LPS-Induced Neuroinflammation, Dopaminergic Dysfunction, and Fatigue-like Behavior in Mice
Source: ACS Omega. 2025 Nov 18;10(47):57568–77. doi: 10.1021/acsomega.5c05266 (PMC12676326; doi:10.1021/acsomega.5c05266)
Supplement: Supplementary file 1 [file ao5c05266_si_001.pdf]

**Supporting Information for Publication**

**Title:** Prior exercise attenuates LPS-induced neuroinflammation, dopaminergic dysfunction, and fatigue-like behavior in mice

**Authors:** Ana Cristina de Bem Alves[1,2], Naiara de Souza Santos[1], Ananda Christina Staats Pires[2], Ana Elisa Speck[1,3], Tatyana Nery[1], Amanda Leite Bastos-Pereira[1], Débora da Luz Scheffer[2], Rui Daniel Prediger[3], Alexandra Latini[2], Aderbal Silva Aguiar Jr[1,2] *

**Affiliations:**

1. Biology of Exercise Lab (Labioex), Department of Physical Therapy, UFSC-Federal University of Santa Catarina, 88905-120, Araranguá, SC, Brazil
2. Laboratory of Bioenergetics and Oxidative Stress (Labox), Department of Biochemistry, UFSC-Federal University of Santa Catarina, 88040-900, Florianópolis, SC, Brazil
3. Experimental Laboratory of Neurodegenerative Diseases (LEXDON), Department of Pharmacology, Federal University of Santa Catarina, 88040-900, Florianópolis, SC, Brazil

**Corresponding author:** Aderbal S. Aguiar Jr., Laboratory of Exercise Biology (LABIOEX), Federal University of Santa Catarina (UFSC), Araranguá, SC, 88905-120, Brazil. Phone: 55-48-3721-6956. Email: aderbal.aguiar@ufsc.br

**ACS Omega – Manuscript ID: ao-2025-05266n.R1**

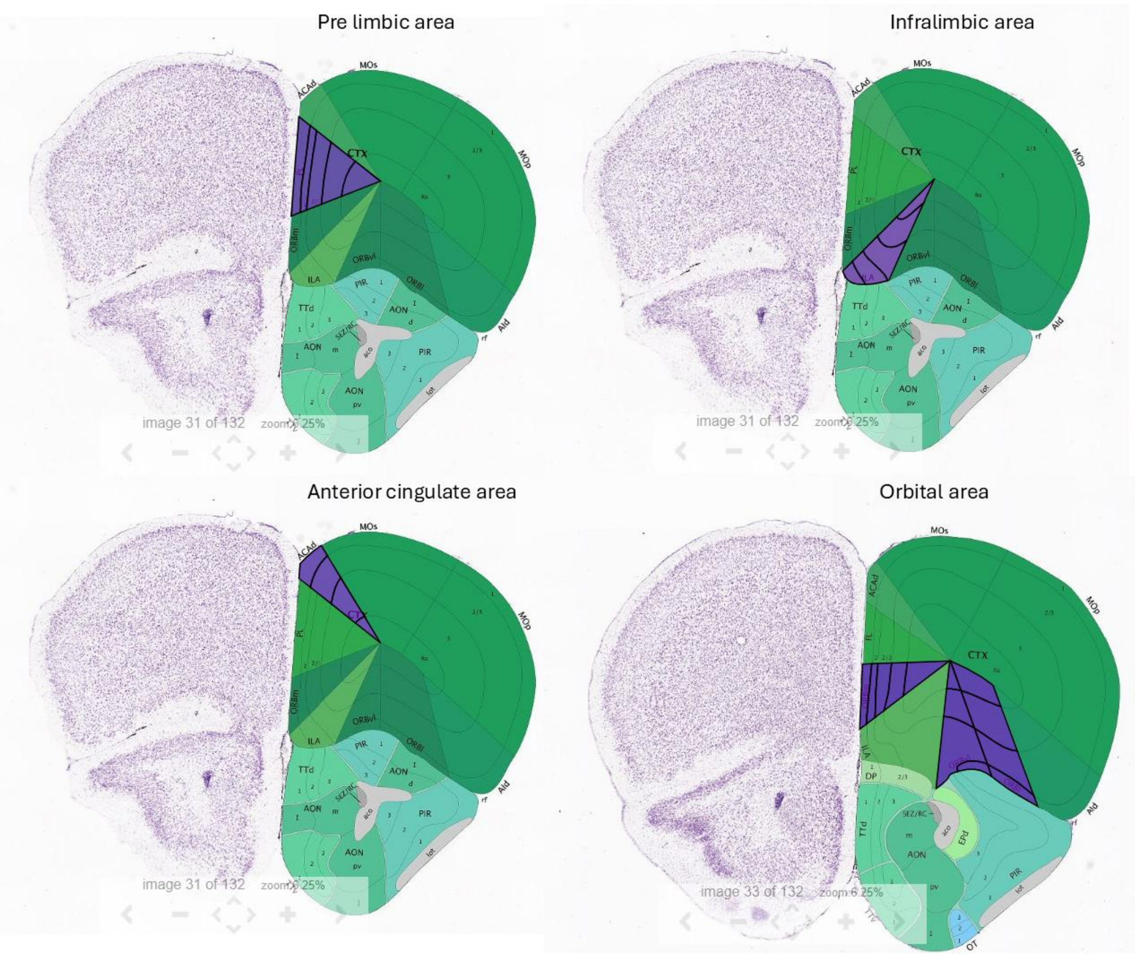


Figure S1. Schematic representation of the dissected prefrontal cortex regions (PrL, IL, ACC, OFC) collected bilaterally for molecular analysis. Image adapted from the Allen Brain Atlas (https://portal.brain-map.org/).

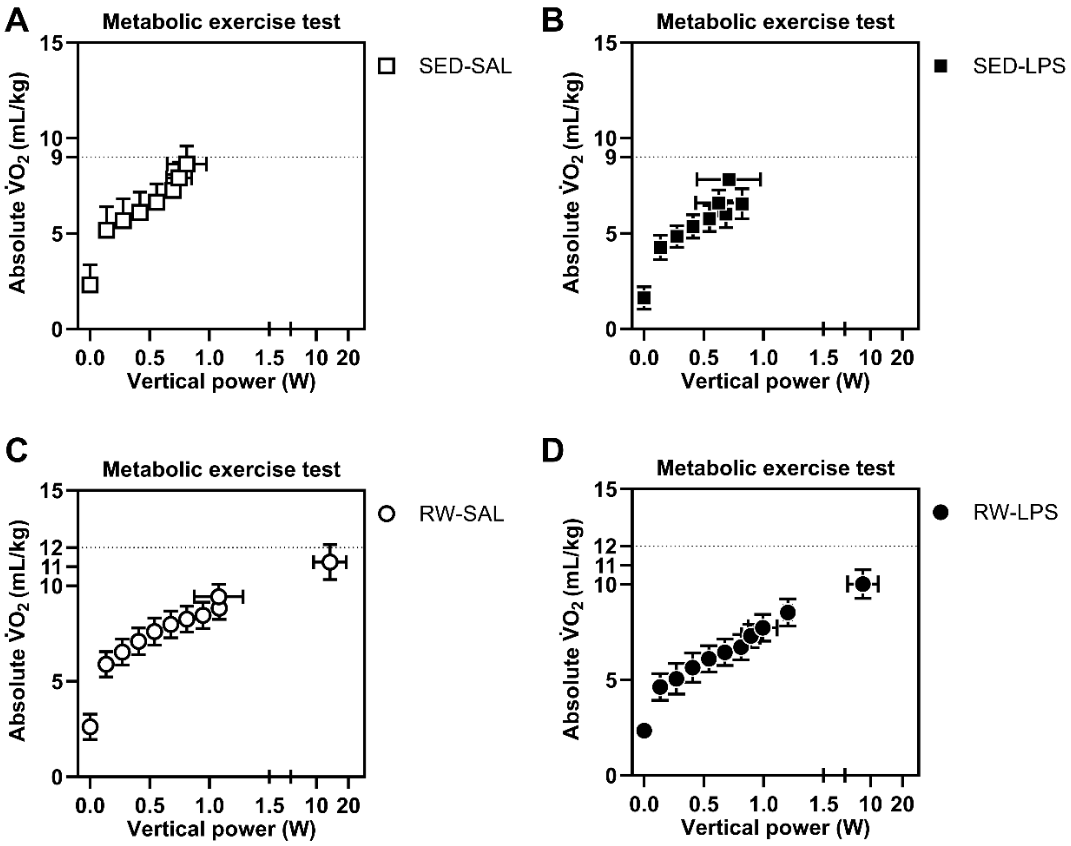


Figure S2. $\dot{V}O_2$ curves by experimental group (SED-SAL, SED-LPS, RW-SAL, RW-LPS) during incremental treadmill test. Data are mean ± SEM, N = 8–10 animals per group.
